# An Evaluation of Knowledge and Comfort in Discussing the Human Papillomavirus (HPV) Vaccine Among a Sample of Physicians Practicing in South Carolina

**DOI:** 10.7759/cureus.45247

**Published:** 2023-09-14

**Authors:** Jessica Diaz Rijo, Jenna Magri, Alexis Stoner, Lisa Carlson, Karen Fradua, Lisa Carroll, David Redden

**Affiliations:** 1 Preventive Medicine, Edward Via College of Osteopathic Medicine, Spartanburg, USA; 2 Epidemiology and Public Health, Edward Via College of Osteopathic Medicine, Spartanburg, USA; 3 Public Health, South Carolina Department of Health and Enivronmental Control, Columbia, USA; 4 Public Health, South Carolina Department of Health and Environmental Control, Columbia, USA; 5 Family Medicine, Edward Via College of Osteopathic Medicine, Spartanburg, USA; 6 Research and Biostatistics, Edward Via College of Osteopathic Medicine, Auburn, USA

**Keywords:** human papillomavirus, hpv-related disease, child/adolescent vaccines, cancer prevention, hpv vaccine

## Abstract

Objective

To determine knowledge and comfort in discussing the human papillomavirus (HPV) vaccine among a sample of physicians practicing in South Carolina.

Methods

This descriptive cross-sectional study utilized a 33-question survey assessing knowledge of HPV, the HPV vaccine, and comfort in discussing associated topics with patients among a sample of physicians across the state of South Carolina. Descriptive and correlational analyses were performed.

Results

Of the total 66 participants, most self-reported having sufficient knowledge about HPV, yet responses to fact-based questions yielded an average score of only 7.03/13. Most felt comfortable discussing HPV, while some reported discomfort discussing sex-related topics (3.6%). A positive significant correlation was determined between having sufficient knowledge of HPV and comfort levels discussing both HPV and sex-related topics ((p-value < 0.001), (p = .0028)), comfort levels discussing HPV and comfort levels discussing sex (p = .0030), and comfort level discussing sex and previous communication training (Mantel-Haenszel chi-square = 0.0447).

Conclusions

The results of this study support the role of future interventions aimed at increasing the HPV knowledge base and training in discussions of sex for providers to help increase HPV vaccination rates in South Carolina.

## Introduction

The human papillomavirus (HPV) is the most common sexually transmitted infection in the world, initially transmitted to teens and individuals in their early twenties [1]. This virus belongs to a family of viruses that infect human epithelial tissues and is classified into high-risk and low-risk types [2]. The most notable high-risk types are HPV-16, 18, 31, and 33, which have been shown to cause cervical, vaginal, vulvar, penile, oropharyngeal, and anal cancers [3]. Low-risk types-most commonly HPV-6 and 11-cause genital warts and are very rarely associated with precancerous lesions. Human papillomavirus is transmitted via skin-to-skin or skin-to-mucosa contact and can be transmitted to others during vaginal, anal, or oral sex, even if the infected partner is asymptomatic. Symptoms often take years to become noticeable, and thus infected individuals most often do not know when they were first infected, making it difficult to track down infected sexual partners [1].

The first HPV vaccine, Gardasil, was released in 2005 [4]. Since that time, there have been three vaccines approved by the Federal Drug Administration, including Gardasil, Gardasil 9, and Cervarix. Gardasil 9 has only been distributed in the United States (U.S.) since 2016. Routine HPV vaccination is recommended for male and female adolescents, starting as early as age nine. Despite the vaccine’s effectiveness at preventing over 90% of HPV-related cancers [5], the rates of HPV vaccination have remained low in the U.S., with only 60% of U.S. adolescents between the ages of 13 and 17 receiving one dose out of the total three doses of the HPV vaccine. The remaining 40% of U.S. adolescents have never received a single dose of the vaccine [3]. As part of the Healthy People 2020 federal program, the U.S. set a goal of 80% HPV vaccination levels for male and female adolescents aged 13-15 years old. As a country, the U.S. did not meet this goal, and South Carolina consistently had one of the lowest rates of HPV vaccination in the country [6-8].

Risk factors associated with consistently low levels of HPV vaccination include male gender, low socioeconomic status, lower education level, Spanish-speaking, Hispanic, non-privately insured, and residence in a rural and/or southern region [9, 10]. In targeting these risk factors, studies have shown the combination of community-level intervention with provider-level intervention is most effective in increasing HPV vaccination coverage [11]. A modifiable risk factor with interventional methods is the role providers play in HPV vaccination. Providers and other healthcare personnel are responsible for initiating and guiding the discussion about HPV and HPV vaccination with patients and their parents. As highly educated and specially trained members of the community, their interactions directly influence whether a child initiates and/or completes the multidose HPV vaccine [10, 12]. Unfortunately, the HPV vaccine is different in many ways from other routine childhood and adolescent vaccinations due to historical marketing strategies and its strong association with being sexually active. These factors often contribute to levels of discomfort among physicians, in addition to feeling ill-equipped to recommend these vaccines effectively [9, 10]. This lack of or ineffective communication therefore plays a large role in preventing the nation from reaching HPV vaccination rate goals [9, 10].

Provider knowledge, communication methods, ability to endorse vaccinations, and comfortability in addressing HPV and sex-related topics all affect discussions of HPV [9, 10, 13]. Patients and their parents have been shown to desire more information about HPV, the vaccine, and associated risks and benefits from their providers, but physicians are often unsure of which information is most important to their patients and which will be most influential in promoting HPV vaccination [14]. Provider’s practice methods, including whether they utilize a less effective risk-stratified versus more effective age-dependent approach to initiate HPV vaccination discussions and whether they discuss HPV vaccination together with or separately from other routine childhood vaccinations, are important determinants in rates of HPV vaccination [9-10]. Finally, previous studies have shown a lack of comfort in discussing sex and sex-related topics such as HPV, and perceived parental hesitancy in agreeing to HPV vaccination often hinders the initiation of a necessary discussion about HPV and the HPV vaccine [9, 15]. Many campaigns and government programs, including Vaccines for Children (VFC), have been developed to increase awareness of and accessibility to HPV vaccination in the public. Vaccines for Children is a federally funded program that provides vaccines at no cost to children who might not otherwise be vaccinated due to their inability to afford these services [16]. Additionally, experts in the field support the need for further interventions for healthcare providers and the role this training may play in increasing HPV vaccination [17]. Research, specifically in South Carolina, has shown that simply being in proximity to providers enrolled in programs such as VFC does not lead to increased HPV vaccination rates [18]. Research directed toward understanding areas of weakness in the provider’s role in HPV vaccination discussions, specifically in South Carolina, is lacking. This study assesses South Carolina providers’ knowledge about HPV, HPV-related diseases, and the HPV vaccine, as well as evaluates their comfort in discussing topics related to HPV and the HPV vaccine, including sex.

## Materials and methods

This project was reviewed and approved as exempt by the Institutional Review Board (IRB) of the Edward Via College of Osteopathic Medicine (VCOM), Spartanburg, USA (record number 2021-034), the South Carolina Department of Environmental Control (SC-DHEC) IRB (IRB.22-001), and the Spartanburg Regional Healthcare System (SRHS) IRB (reference number 1951075). Our study did not require ethical board approval because the survey used for data collection was anonymous, and therefore no form of identification was acquired from any participants in this study.

This cross-sectional study utilized an anonymous survey distributed to South Carolina pediatricians and family medicine providers. The survey was developed and validated in coordination with an expert panel consisting of members of the South Carolina Department of Health and Environmental Control Adolescent Immunization Task Force. Questions were developed using evidence-based sources and reviewed by the expert committee for accuracy and construct validity. The survey consisted of three questions about current clinical practices related to HPV vaccination, three questions about participation in VFC program and the availability of onsite HPV vaccines at their practice, one question as a geographic data point, thirteen questions to evaluate provider knowledge of HPV, HPV-related diseases, and the HPV vaccine, 10 questions asking providers to self-rank their level of knowledge of HPV and the HPV vaccine, their comfort level in discussing the HPV vaccine with patients and their parents, and their ability to provide a strong endorsement of vaccinations including the HPV vaccine, and one question about previous formal training on discussing sex and sex-related topics. The survey concluded with a section for respondents to submit comments about topics on which they would be interested in receiving further training or information.

The survey was created using Qualtrics (Qualtrics, Provo, UT, USA). and distributed via email to 444 physicians using a convenience sampling approach from several distribution lists reaching physicians across the entire state of South Carolina. This included 367 physicians practicing in South Carolina who are registered with the VFC program. It was initially distributed to this cohort in February 2022 and remained open for nine months. The survey was also distributed to 77 physicians identified as pediatricians and family medicine providers associated with SRHS. It was initially distributed in September 2022 and remained open for roughly two months. The survey was open for differing time periods for responses from the two cohorts due to delays in project approval and distribution coordination at SRHS. Subsequently, the link for the survey was distributed and made available for submission, beginning later, to those at SRHS rather than those members of VFC. The survey was closed to all in November 2022.

Statistical analyses began by summarizing the proportion of respondents selecting specific responses. For questions that could be categorized as choosing a correct vs. incorrect answer, we summarized proportions as percent answering correctly. We utilized the method proposed by Wilson to provide 95% confidence intervals for these proportions [19]. Wilson’s method was chosen as the statistical literature indicates this approach provides the most appropriate coverage when proportions are estimated at the boundaries of 0 or 1 [19]. Additionally, we sought to evaluate how providers determine with which patients and families to discuss HPV. We compared an age-dependent model in which providers address HPV vaccination with every patient once they reach a certain age with a risk-stratification model in which providers address HPV vaccination with patients that meet certain criteria. The examination of the data began with the calculation of descriptive statistics. Specifically, for continuous and semi-continuous measures, such as the percentage of HPV knowledge questions answered correctly or the sum of Likert scale questions, sample means and standard deviations were calculated to measure the central location and spread. For categorical measures, such as the COVID-19 vaccination status of parents and children, frequency and proportions were summarized. Chi-square tests of association were used to test the relationships among categorical variables. To measure correlations between scores, Spearman’s correlation was calculated, and a hypothesis test was conducted using the null hypothesis that the true correlation was zero. To compare groups on sums of Likert scale questions, the non-parametric tests Wilcoxon rank-sum (comparing two groups) and Kruskal-Wallis (more than two groups) were used. Spearman correlations, Wilcoxon rank-sum, and Kruskal-Wallis were chosen due to the ordinal nature of the data. All analyses used a two-tailed Type I error rate of 0.05 and were conducted using SAS 9.4 (SAS Institute, Cary, NC, USA).

## Results

**Figure 1:**
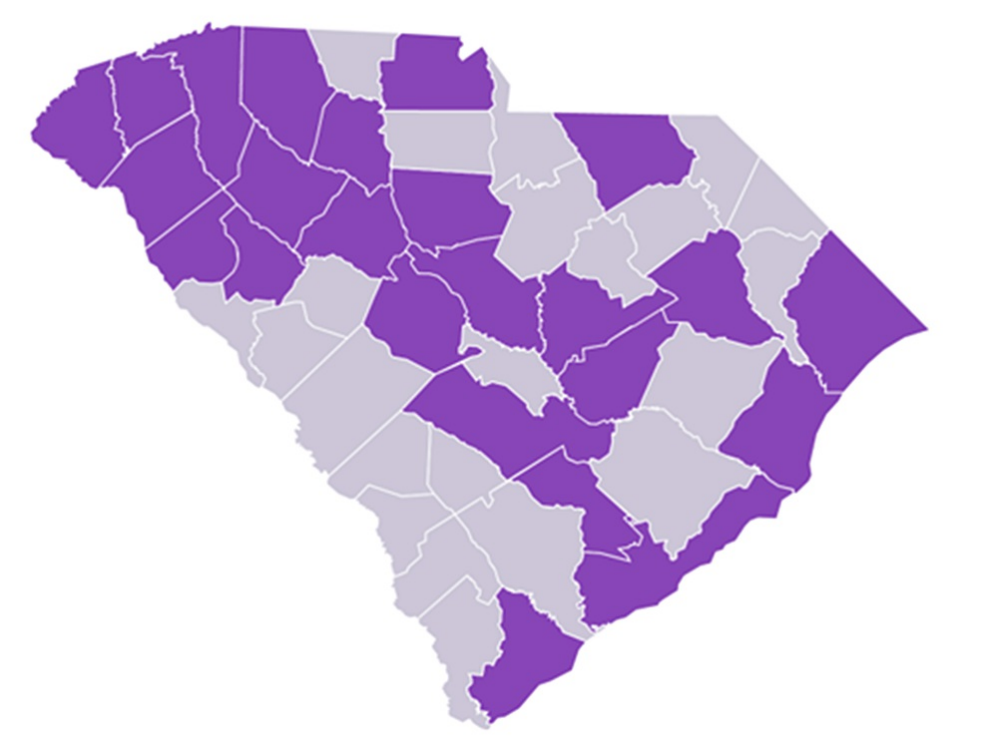
County map of South Carolina Participants in the survey submitted geographic data about the county they practiced in. Counties identified as purple on the map represent counties from which responses were received. No responses were received from gray counties. This figure was originally developed by the authors.

A total of 65 physicians practicing in South Carolina and one physician practicing in North Carolina participated in the research. The sample included a representative distribution of South Carolina counties, including Abbeville, Anderson, Beaufort, Charleston, Chesterfield, Clarendon, Dorchester, Fairfield, Florence, Georgetown, Greenville, Greenwood, Horry, Laurens, Lexington, Newberry, Oconee, Orangeburg, Pickens, Richland, Spartanburg, Sumter, Union, and York (Figure 1).

HPV vaccine introduction and approach methods

The youngest age at which providers initiate discussion of the HPV vaccine is nine years old (19, 37.3%), though the majority (25, 49%) do so at the Centers for Disease Control and Prevention (CDC)-recommended age of 11-12 years old (Table 1).

**Table 1:** Survey questions about current practices regarding HPV vaccination HPV: human papillomavirus; STIs: sexually transmitted infections

Question Number	Survey Question Prompt with Possible Responses	Frequency	Percentage
1.	Do you discuss HPV vaccination with all patients who are eligible?		
	Yes	10/65	15.4%
	No	55/65	84.6%
a.	If yes, at what patient age/age range do you discuss HPV vaccination?		
	9 years old	19/51	37.3%
	10 years old	4/51	7.8%
	11 years old	22/51	43.1%
	12 years old	3/51	5.9%
	13 years old	2/51	3.9%
	Other	1/51	2%
b.	If no, how do you determine which patients to discuss the HPV vaccine with?		
	Female gender	9/9	100%
	Male gender	8/9	88.9%
	Age: 11-12 years old	8/9	88.9%
	Age: 13-17 years old	8/9	88.9%
	Age: 18 years old and above	3/9	33.3%
	Insurance status	2/9	22.2%
	History of STIs	1/9	11.1%
	Perceived sexual activity	1/9	11.1%
	Other (free text): Unvaccinated status	1/9	11.1%
	I do not discuss the HPV vaccine with my patients	0/9	0%
2.	Do you discuss HPV vaccinations with other routine childhood vaccinations or separately from other routine vaccinations?		
	I discuss the HPV vaccine with other childhood vaccinations	53/60	88.3%
	I discuss the HPV vaccine separately	5/60	8.3%
	Other (free text): I only discuss HPV because it is the only vaccine I give	1/60	1.7%
	Other (free text): Both together and separately	1/60	1.7%
	I do not discuss the HPV vaccine with my patients	0/60	0%
3.	Do you generally recommend administering HPV vaccinations the same day you have the discussion with the patient and their parents/guardians?		
	Same day	59/60	98.3%
	Discuss and vaccinate at different appointments	1/60	1.7%
	I do not discuss the HPV vaccine with my patients	0/60	0%
4.	Do you administer HPV vaccines in your office?		
	Yes	53/55	96.4%
	No	2/55	3.6%
5.	Does your office generally have HPV vaccines on-site to provide to patients?		
	Yes	53/55	96/4%
	No	2/55	3.6%

The overwhelming majority (55, 84.6%) utilize an age-dependent approach, discussing the HPV vaccine with each patient at a specific age. Of those that utilize a risk-stratification approach, the most important factors in determining which patients to discuss the HPV vaccine included female gender (nine, 100%), male gender (eight, 88.9%), age (seven, 75%), and insurance status (two, 22.2%). It was found that 53 (88.3%) of providers discuss the HPV vaccine together with other childhood vaccinations, while five (8.3%) discuss it separately, and after having that discussion and obtaining patient consent, 59 (98.3%) administer the HPV vaccine that same day. However, there was no significant correlation between responses to whether a provider discussed the HPV vaccine with every patient and whether or not they discussed the HPV vaccine with other childhood vaccinations (p = 0.58).

Human papillomavirus & HPV vaccine knowledge

The overall knowledge score for each participant was calculated based on the number of correct responses out of the 13 fact-based questions on HPV, HPV-related diseases, and the HPV vaccine posed in the survey. The mean score for all participants was 7.03/13, with a standard deviation of 1.91. The results were then broken down into percentiles, with minimum (0/14), 25th percentile (6.0/13), median (7.0/13), 75th percentile (8.0/13), and maximum (11/13).

The majority of participants knew that HPV has been associated with cancers of the cervix, vagina, vulva, penis, anus, and oropharynx, though only 17 (28.8%) correctly selected all these cancers (95% CI (18.8%, 41.4%)) (Table 2).

**Table 2:** Fact-based multiple choice questions evaluating the knowledge level of HPV, HPV-related diseases, and the HPV vaccine Statistical parameters include N%, and 95% confidence intervals (CI) to compare correct vs. incorrect answer choices. HPV: human papillomavirus

Question Number	Survey Question Prompt with Possible Responses	Frequency	Percentage	95% CI
1.	How is HPV transmitted?			
	Direct skin-to-skin contact	35/59	59.3%	[46.6%,70.9%]
	Bodily fluids	24/59	40.7%	
	Respiratory droplets	0/59	0%	
	Fecal-oral	0/59	0%	
2.	Are the main strains associated with cancer covered by the HPV vaccine?			
	Yes	59/59	100%	[93.9%, 100%]
	No	0/59	0%	
3.	What percentage of the population is estimated to be infected with HPV at some point in their life?			
	85%	19/59	32.2%	[21.7%, 44.9%]
	70%	18/59	30.5%	
	50%	15/59	25.4%	
	25%	6/59	10.2%	
	10%	1/59	1.7%	
4.	HPV can cause which of the following diseases? (multiple answers may be selected)			
	Selected combination of ALL correct responses	17/59	28.8%	[18.8%, 41.4%]
	Selected at least one correct response	59/59	100%	
	Cervical cancer	59/59	100%	
	Anal cancer	57/59	96.7%	
	Throat cancer	57/59	96.7%	
	Penile Cancer	54/59	91.5%	
	Squamous cell carcinoma	18/59	30.5%	
	Basal cell carcinoma	0/59	0%	
	Sepsis	0/59	0%	
	AIDS	0/59	0%	
5.	Which of the following tests is used to screen for HPV infection?			
	DNA test	34/59	57.6%	[44.9%, 69.4%]
	Cervical biopsy	15/59	25.4%	
	Antibody-antigen assay	8/59	13.6%	
	Wet mount	2/59	3.4%	
6.	Of the 45,300 HPV-associated cancer cases estimated to occur in the US each year, what percentage are in women compared to men?			
	56% in women; 44% in men	19/59	32.2%	[21.7%, 44.9%]
	73% in women; 27% in men	16/59	27.1%	
	84% in women; 16% in men	13/59	22.0%	
	69% in women; 31% in men	11/59	18.6%	
7.	How many HPV vaccines are currently in clinical use in the United States?			
	1	19/59	32.2%	[21.7%, 44.9%]
	2	29/59	49.2%	
	3	11/59	18.6%	
8.	What type of vaccine is the current HPV vaccine?			
	Recombinant	49/56	87.5%	[76.4%, 93.8%]
	mRNA	4/56	7.1%	
	Live attenuated	3/56	5.4%	
	Toxoid	0/56	0%	
9.	Which strains of HPV does the currently available vaccine protect against?			
	6, 11, 16, 18, 31, 33, 45, 52, 58	44/54	81.5%	[69.2%, 89.6%]
	6, 11, 16, 18	9/54	16.7%	
	16, 18, 31, 33	1/54	1.9%	
	6, 11	0/54	0%	
10.	Which of the following is a contraindication for the HPV vaccine currently in clinical use (Gardasil-9)?			
	Allergy to yeast	12/13	92.3%	[66.7%, 98.6%]
	Allergy to latex	1/13	7.7%	
	Mildly ill patient	0	0%	
	None of the above	0	0%	
11.	What are the most common adverse effects of the HPV vaccine Gardasil-9?			
	Selected ALL correct answers	3/57	5.3%	[1.8%, 14.4%]
	Selected at least one correct answer	56/57	98.2%	
	Injection site pain	55/57	96.5%	
	Injection site erythema	41/57	71.9%	
	Injection site swelling	40/57	70.2%	
	Nausea	14/57	24.6%	
	Headache	19/57	33.3%	
	Fever	14/57	24.6%	
12.	Is the HPV vaccine single-dose or multiple-dose?			
	2 or 3 doses	56/56	100%	[93.9%, 100%]
	1 dose	0/56	0%	
	Single dose + a booster in 15 years	0/56	0%	
13.	At what age does the CDC recommend initiating the HPV vaccine?			
	11-12 years old	56/56	100%	[93.9%, 100%]
	12-15 months	0/56	0%	
	4-6 years old	0/56	0%	
	15-18 years old	0/56	0%	

Roughly a third (19, 32.2%) correctly answered that 85% of people are estimated to be infected with HPV at some point in their life (95% CI (21.7%, 44.9%)). The same percentage recognized the correct distribution of HPV-associated cancer cases in the U.S. as 56% for women and 44% for men (95% CI (21.7%, 44.9%)). Only 35 (59.32%) correctly answered the mode of HPV transmission as direct skin-to-skin contact, with 24 (40.7%) incorrectly selecting bodily fluids (95% CI (46.6%, 70.9%)). Similarly, 34 (57.6%) knew that HPV screening is performed using DNA tests (95% CI (44.9%, 69.4%)).

Out of all respondents, 19 (32.2%) knew there is only one vaccine, Gardasil-9, currently in clinical use in the U.S. (95% CI (21.7%, 44.9%)). The majority (49, 87.5%) correctly identified it as a recombinant vaccine (95% CI (76.4%, 93.8%)). All providers (56, 100%, 95% CI (93.6%, 100%)) correctly responded that the HPV vaccine is a multidose vaccination that provides protection against the main strains associated with HPV-related cancers. and also correctly identified the age of initiation of the vaccine regimen according to the CDC as 11-12 years old (95% CI (93.6%, 100%)). For questions targeting contraindications of the vaccine, only a small portion of people provided a response. The proper interpretation of this data point is unclear, but it was inferred that a non-response was equivalent to not knowing the correct answer. Of those that did provide a response, 12 (92.3%) knew an allergy to yeast was the correct contraindication (95% CI (66.7%, 98.6%)). When asked about adverse effects, only three (5.3%) selected a combination of all correct answers, and 56 (98.2%) identified at least one of the most common adverse effects.

Self-assessment of the knowledge level of HPV, comfort level discussing HPV, and comfort level discussing sex-related topics

**Figure 2:**
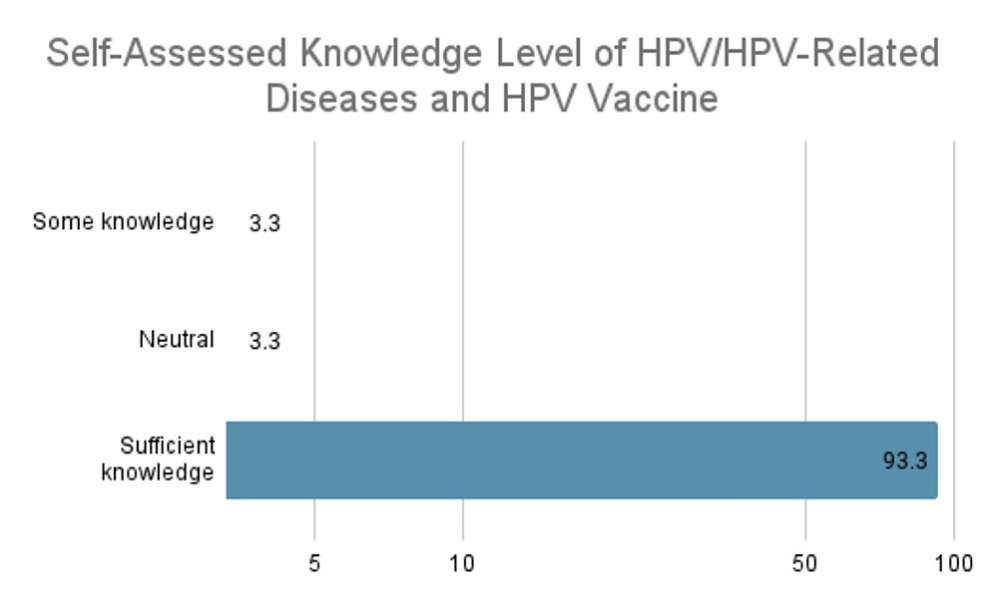
Self-assessed knowledge level of HPV/HPV-related diseases and the HPV vaccine HPV: human papillomavirus

The distribution of responses regarding the self-assessed knowledge level of HPV/HPV-related diseases and the HPV vaccine was similar and therefore collapsed into one sum representing the general knowledge level of HPV (Figure 2).

Two participants (3.3%) reported having some knowledge, two (3.3%) reported feeling neutral, and 56 (93.3%) reported having sufficient knowledge in their discussions of the subject with patients or parents.

**Figure 3:**
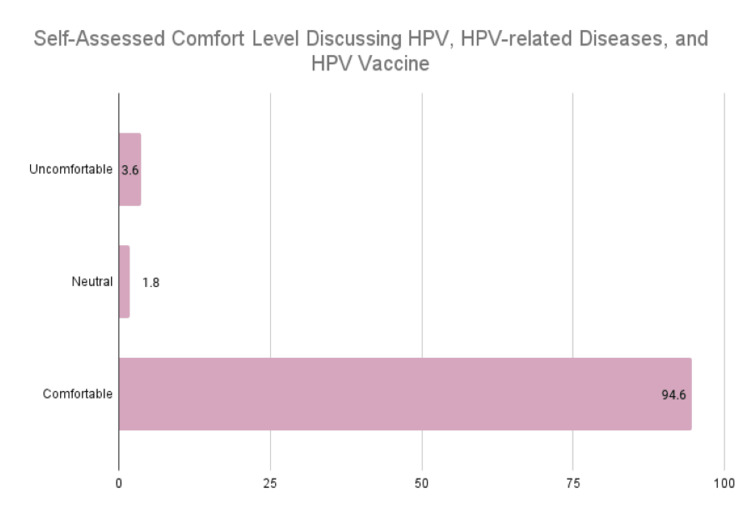
Self-assessed comfort level discussing HPV, HPV-related diseases, and the HPV vaccine HPV: human papillomavirus

The distribution of responses regarding comfort level in discussions of HPV, HPV-related diseases, and the HPV vaccine separately was similar and therefore collapsed into one sum representing general comfort level discussing HPV (Figure 3).

Two participants (3.6%) reported feeling uncomfortable, one (1.8%) reported feeling neutral, and 53 (94.6%) reported feeling comfortable in these discussions.

**Figure 4:**
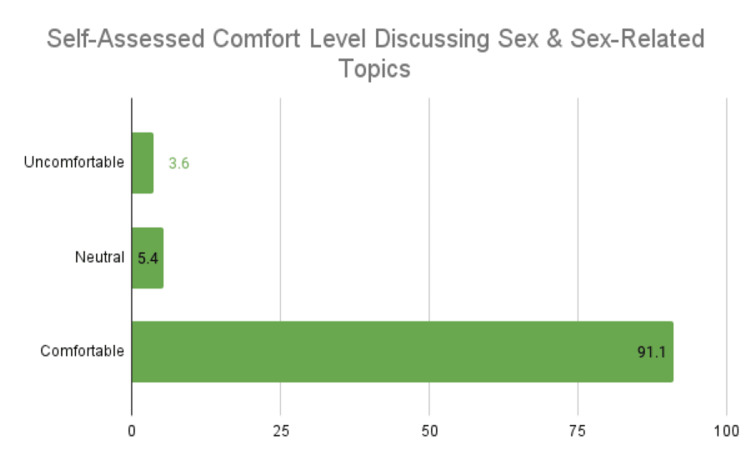
Self-assessed comfort level discussing sex and sex-related topics

Provider comfort level in discussing sex and sex-related topics showed that 3.6% were uncomfortable, 5.4% were neutral, and 91.1% were comfortable (Figure 4).

In addition, our results showed that there is a significant association between previous formal training in communication on sex-related topics and reported comfort level in discussing sex (Mantel-Haenszel chi-square = 0.045).

Correlation between HPV/HPV vaccine knowledge and comfort level

We found a positive correlation between overall performance on the fact-based quiz questions, or knowledge score, and comfort level in discussing HPV. Although a positive correlation was noted, the correlation was not statistically significant (p = 0.23) (Table 3).

**Table 3:** Correlational analysis results Chi-square tests of association were used to test the relationships among categorical variables. Spearman's correlations were chosen for ordinal data. All analyses used a two-tailed type I error rate of 0.05. HPV: human papillomavirus; VFC: Vaccines For Children

Variable 1	Variable 2	Statistical Analysis	Statistically Significant
Statistically significant positive correlations			
Self-assessed sufficiency of HPV knowledge	Comfort level discussing HPV	Spearman's correlation coefficient = 0.54 p-value < 0.001	Yes
Self-assessed sufficiency of HPV knowledge	Comfort level discussing sex-related topics	Spearman's correlation coefficient = 0.62 p-value = 0.0028	Yes
Comfort level discussing HPV	Comfort level discussing sex-related topics	Spearman's correlation coefficient = 0.87 p-value = 0.0030	Yes
Previous formal training in the communication of sex-related topics	Comfort level discussing sex-related topics	Mantel-Haenszel chi-square = 0.045	Yes
Statistically insignificant positive correlations			
Knowledge score	Comfort level discussing HPV	Spearman's correlation coefficient = 0.16 p-value = 0.23	No
Knowledge score	Self-assessed sufficiency of HPV knowledge	Spearman's correlation coefficient = 0.22 p-value = 0.10	No
Knowledge score	Comfort level discussing sex-related topics	p-value = 0.56	No
No correlation and insignificant			
VFC participation	Self-assessed sufficiency of HPV knowledge	p-value = 0.14	No
VFC participation	Comfort level discussing HPV	p-value = 0.09	No
VFC participation	Knowledge score	p-value = 0.45	No
VFC participation	Age-dependent v. risk stratification model of which patients to discuss HPV	p-value = 0.15	No
VFC participation	Discussion of HPV with childhood vaccines or separately	p-value = 1.00	No
Age-dependent v. risk stratification model of which patients to discuss HPV	Discussion of HPV with childhood vaccines or separately	p-value = 0.58	No

Similarly, a positive correlation was found between knowledge score and self-reported knowledge sufficiency, though this was not statistically significant (p = 0.10). In addition, we found a statistically significant (p < 0.001) positive correlation between self-reported knowledge sufficiency and comfort when discussing HPV. The correlation between knowledge score and comfort in discussing sex or sex-related topics, comfort discussing HPV, and self-reported knowledge level of HPV when comparing the knowledge score with comfort in discussing sex had a weak positive correlation and a nonsignificant p-value (p = 0.56). However, our analysis did show a positive and significant correlation between self-assessed sufficient knowledge of HPV and a higher level of comfort discussing sex-related topics (p =.0028). In addition, there was a positive and significant correlation between providers who reported comfortability in discussing HPV and high levels of comfortability in discussing sex with patients (p = 0.0030). There was a positive but statistically insignificant correlation between knowledge score and responses to either self-reported sufficiency of knowledge of HPV or comfort level in discussing HPV in general.

Vaccines for Children participation

Of note, 49 respondents (74%) reported being a VFC program provider. There was no correlation between participation status in the VFC program and response to current HPV practices, including whether or not the provider discusses the HPV vaccine with every patient and whether or not they discuss the HPV vaccine with other childhood vaccinations (p = 0.15; p = 1.00, respectively) (Table 3).

We compared whether the distribution of knowledge scores differed based on whether the respondent was associated with the VFC program. We found there is no significant difference in knowledge score between VFC program participants and non-VFC program participants (median knowledge score 8 vs. 7, respectively) (p = 0.45). There was no significant correlation between participation in VFC and the reported sufficiency of knowledge of HPV or comfort level in discussing HPV in general (p = 0.14; p = 0.09, respectively).

HPV vaccine endorsement

**Figure 5:**
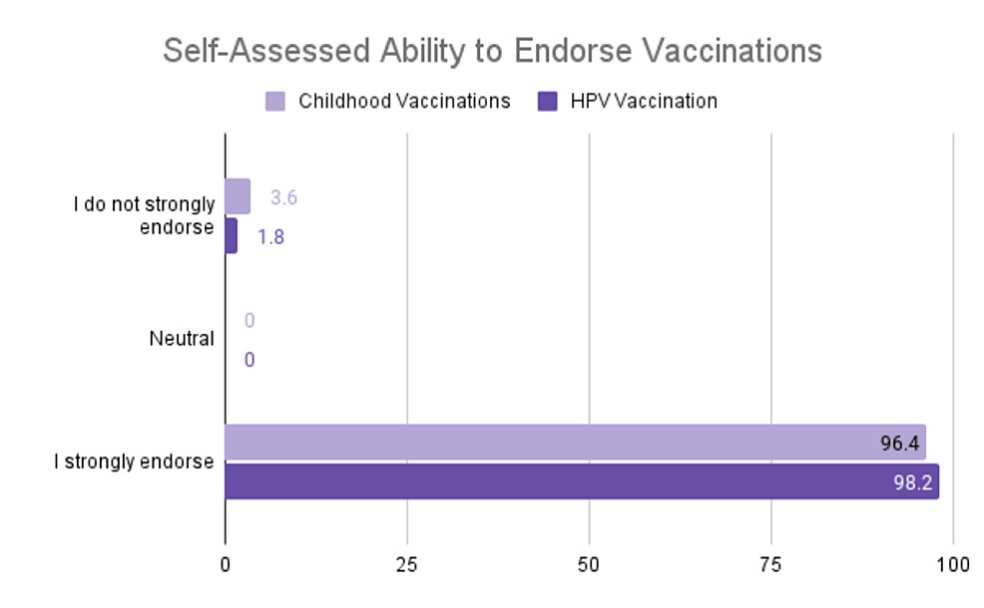
Self-assessed ability to endorse vaccinations

Our results showed that 98.2% of respondents strongly endorse the HPV vaccine and 96.4% strongly endorse childhood vaccination (Figure 5).

However, 3.6% do not strongly endorse childhood vaccinations, and 1.8% do not strongly endorse HPV vaccinations.

## Discussion

The results support that the majority of South Carolina physicians who participated in our study are utilizing the more effective age-dependent strategy of approaching HPV vaccination with their patients. Knowledge level self-assessments showed discordance between providers’ perceived knowledge level of HPV and the HPV vaccine with the percentage of correct answers on fact-based questions covering the same topics. Despite most participants feeling comfortable in these conversations about HPV vaccination, there is a portion reporting discomfort, especially when approaching discussions of reproductive health. Additionally, an analysis comparing the responses of VFC participants to those of non-participants showed no significant difference between these groups.

Previous studies have indicated that an age-dependent model of determining which patients to discuss HPV vaccinations is more effective and associated with higher rates of HPV vaccination than employing a risk-stratification model in which HPV vaccine discussions are determined based on the presence or absence of certain patient criteria [9, 10]. Almost all participants reported using an age-dependent model, addressing HPV vaccination as early as nine years old, though age 11-12 was the most popular response. This shows that providers adhere to the CDC guidelines of beginning this vaccination regimen at 11-12 years old, with many beginning even earlier. Criteria for those who utilize risk stratification were consistent with previous studies showing gender as the most important factor [9, 10]. Our findings showed a slight preference for females compared to males, which could reflect the lower numbers of males who are HPV vaccinated in South Carolina.

Regarding the HPV vaccine, most providers felt they had sufficient knowledge, but the knowledge score determined by fact-based questions contradicted this subjective measure on certain topics. The mean correct answers were just over 50%. Importantly, many of the topics of concern might come up in discussions of HPV vaccination with patients and their parents, such as virus transmission, associated cancers, and the prevalence of HPV infection in the population. This further highlights the incongruity between perceived provider knowledge and actual HPV vaccine knowledge. Interestingly, the data show higher comfort levels in discussions of HPV are correlated with a higher self-perceived knowledge level of HPV but not necessarily an objective knowledge level based on the determined knowledge score.

In order to have a productive and fruitful discussion about the HPV vaccine with patients and their parents or guardians, providers need more than just sufficient knowledge. They also need to feel comfortable approaching such a topic that is highly connected to sex and reproductive health with very young patients [20]. Additionally, providers must be skilled at endorsing vaccinations in general, including other routine childhood vaccinations [9]. Overall, most respondents felt comfortable discussing HPV; however, a portion of providers reported discomfort with initiating conversations about sex-related topics, which is an important factor for HPV vaccine endorsement [20]. This was further reflected at the conclusion of the survey when prompted to submit topics of interest for training and approaching discussions about sex was the most common response (Table 4). 

**Table 4:** Free-text response at the conclusion of the survey HPV: human papillomavirus; MSM: men who have sex with men

Are there any topics related to HPV, HPV-related diseases, HPV vaccination, discussing sex, and/or any other related topics that you would like further training or information on?
Discussing sex with parents, how to supply the vaccine in my practice, what resources are available, HPV-related diseases, a global brush-up, indications for HPV vaccines in adults, when to recommend HPV vaccines in the MSM patient population, discussing sex in general, discussing sex with adolescents of varying ages and levels of maturity

Given that there is a statistically significant correlation between higher levels of comfort in discussions of sex-related topics and both a higher self-assessed knowledge level of HPV and previous formal training in patient communication regarding sex, this supports the role of future interventions aimed at increasing HPV and HPV vaccine knowledge bases and training in discussions of sex with patients and their parents, particularly how to discuss sex and reproductive health issues with patients of a variety of ages and maturity levels with the intent to facilitate the introduction of the HPV vaccine. This is important to highlight, as only 51.8% of respondents reported receiving formal training on these topics before (Table 5).

**Table 5:** Survey questions about previous formal communication training for sex-related discussions CME: continuous medical education; ACS: American College of Surgeons; DHEC: Department of Health and Environmental Control; IUD: intrauterine device

Survey Question Prompt with Possible Responses	Frequency	Percent
Have you previously received formal communication training for discussing sex and sex-related topics with your patients and their parents/guardians?		
Yes	29/56	51.8%
No	27/56	48.2%
If yes, what form of training did you receive?		
The free text responses collected included the following: CME, didactics, during residency, fellowship training, formal training from infectious disease attending, ACS, graduate courses, preparation for teaching sex education, lectures, medical school, webinars, articles, workshops, conference lectures, online resources, DHEC Preventive Health Training, Nexplanon/IUD pharmaceutical training		

If these factors can be targeted to increase overall comfort levels in these discussions, it could help increase HPV vaccination rates in the state.

Looking forward, future studies could compare the HPV vaccination rates before and after providers have completed HPV training to determine if training has affected the levels of HPV vaccination in South Carolina. Another factor to consider is religion and whether it plays a major role in discussions of HPV and other sexually transmitted diseases, as this was not addressed in our study. Literature has shown that states with more religious influence have decreased vaccination rates [8]. Given South Carolina’s significant number of religious exemptions from vaccinations, this could be an important factor to consider when creating provider education training programs, both from the provider's and patient's perspectives [21]. Finally, future studies should also consider a more in-depth analysis of the discomfort physicians experience when discussing sex-related topics with their patients. This can help improve future training efforts and gain a better understanding of the limitations of current patient care.

Limitations

A potential limitation of this study is that the survey included a small population size (66 respondents). Similarly, we only received responses from 24 of the 43 counties in South Carolina. Another limitation is that the study is solely based on the participants’ responses. If a participant chose not to share their opinions or pick answers that they did not necessarily agree with, then the data would not be entirely accurate. In addition, participants may have misinterpreted a question and, therefore, did not answer the question correctly given the style of rank-based questions. The majority of respondents were part of the VFC program. Their involvement could potentially influence a provider’s ability to endorse the HPV vaccine as opposed to those that are not part of the program, as the program makes access to the vaccine easier and therefore administration more efficient.

## Conclusions

South Carolina HPV vaccination rates are consistently below the national average. Within the constraints of the study, it appears that providers are using recommended strategies when approaching patients about the HPV vaccine. However, knowledge of the disease and level of comfort discussing the vaccine and topics of HPV are variable. Using the results of this study, we hope to better direct and provide more targeted training and/or educational materials for the providers in South Carolina in relation to HPV vaccination. It is the hope that improving providers' knowledge and comfort level will better assist them in providing care and ultimately increase overall HPV vaccination rates, therefore decreasing the burden of disease caused by HPV.
